# Impact of HIV-1 subtypes on gross deletion in the *nef* gene after Korean Red Ginseng treatment

**DOI:** 10.1016/j.jgr.2022.02.005

**Published:** 2022-02-26

**Authors:** Young-Keol Cho, Jung-Eun Kim, Jinny Lee

**Affiliations:** Departments of Microbiology, University of Ulsan College of Medicine, Asan Medical Center, Seoul, 05505, Republic of Korea

**Keywords:** Gross deletion, HIV-1 subtype B, Korean Red Ginseng, *Nef* gene, Non-B subtypes

## Abstract

**Background:**

The number of primary human immunodeficiency virus (HIV)-1 non-B subtype infections (non-B) and that of reports regarding the differences in the pathogenesis of subtype B and non-B infections are increasing. However, to the best of our knowledge, there have been no reports on gross deletion in the *nef* gene (gΔ*nef*) in non-B infections.

**Methods:**

To determine whether there is a difference in the change in CD4^+^ T cells after treatment with Korean Red Ginseng (KRG) between patients with subtype B and non-B infections, we retrospectively analyzed and compared the annual decrease in CD4^+^ T cells (AD) and the proportion of gΔ*nef* in 77 patients who were followed for more than 10 years in the absence of combination antiretroviral therapy.

**Results:**

Overall, AD was significantly faster in patients with non-B infections than in those with subtype B infections. Survival analysis showed that the survival probability was significantly higher in subtype B than in non B-infected patients. These differences mainly resulted from significant differences in the amount of KRG and age. In the patients treated with KRG, there was a significant correlation between the amount of KRG and the AD in both subtypes. Interestingly, there was a significant correlation between the amount of KRG and the proportion of gΔ*nef* in patients infected with subtype B, but not in those infected with non-B. The same phenomenon was observed when the KRG dose was adjusted.

**Conclusion:**

Our results suggest that non-B may be biologically more stable than subtype B.

## Graphical abstract

**Figure:**
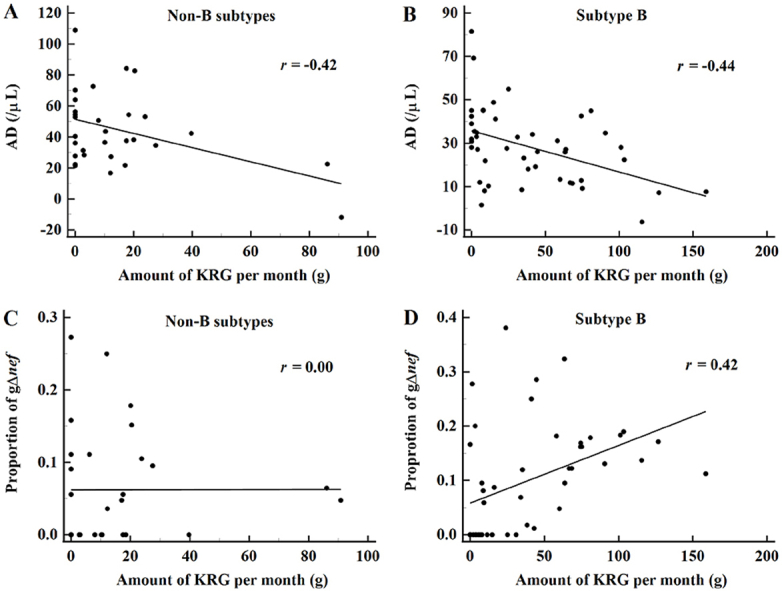

## 1 Introduction

The high viral diversity of human immunodeficiency virus (HIV)-1 has possible implications for the differential rates of disease progression, responses to combination antiretroviral therapy (ART), and the development of a vaccine; however, there are confounders, such as access to medical care, nutritional status, socioeconomic level, host genetic factors, and mode of viral transmission [[1], [2], [3]]. HIV-1 subtype B is the most common subtype in developed countries, and accounts for approximately 11%–12% of the global epidemic [4]. Primary HIV-1 infections with various non-B subtypes (hereafter called non-B) are increasing in developed countries [[4], [5], [6]]. There have been reports on the difference in disease progression among the patients infected with non-B [[7], [8], [9]]. Moreover, there have been reports regarding the differences in the replicative capacity [8,10] and down-modulation of the human leukocyte antigen class I and CD4 receptor [11] between the viral subtypes. In particular, patients with subtype D infections show a faster rate of CD4^+^ T cell decline than that in patients infected with subtype B [12,13]. Despite the different demographic characteristics, including the mode of transmission in Korea (Table 1), to the best of our knowledge, there have been no reports on the differences in disease progression between subtype B and non-B.

**Table 1:** Characteristics of 77 patients infected with human immunodeficiency virus-1 subtype B or non-B subtypes and treated with KRG.

Characteristic	Subtype B	Non-B subtypes	*P*-value
No. of patients	46	31	
Year of diagnosis			
1986–1990	14	17	<0.05
1991–1995	27	14	
1996–2000	4	1	
Sex ratio (M:F)	39:7	27:5	
Age at diagnosis (yr)	25 ± 9	32 ± 7	<0.001
Type of work			
Overseas sailors and their spouses	4	29	<0.0001
Sex worker	2	0	
Others	40	3	<0.0001
Mode of transmission			
Heterosexual contact	9	30	<0.0001
Men who have sex with men	23	1	<0.0001
Transfusion or blood product	14	0	<0.001
Follow-up since diagnosis (months)	170 ± 41	151 ± 34	
No. of patients treated with KRG	38	19	<0.05
Total amount of KRG administered (g)	6816 ± 7303	2408 ± 4930	<0.01
Monthly amount of KRG (g)	39 ± 40	14 ± 22	<0.01
No. of patients treated with zidovudine	11	11	
Plasma RNA (copy/mL) at baseline	13,673 ± 18,551	28,156 ± 53,509	
First CD4^+^ T cell (/μL)	546 ± 284	678 ± 233	<0.05
Last CD4^+^ T cell (/μL)	191 ± 205	159 ± 138	
Interval from first to last CD4^+^ T cells (months)	163 ± 40	161 ± 61	
Annual decrease in CD4^+^ T cells (/μL)	28 ± 17	45 ± 24	<0.001

KRG, Korean Red Ginseng.

This suggests that delta-nef played a major role in disease progression.

In the control group that did not take red ginseng, the delt-nef ratio was significantly higher in the patients followed for more than 10 years than in the follow-up group for less than 10 years.

This seems to have contributed to long-term survival by slowing the annual decrease in the number of CD4^+^ T cells.

This seems to be a defective gene contributed to the long-term survival and slower CD4 + T cells, open reduction in the number.

The HIV-1 *nef* gene is important for the maintenance of high viral loads and is critical for progression to acquired immunodeficiency syndrome (AIDS) [14,15]. Therefore, it is considered the main determinant of virulence [16]. Many studies have shown that long-term slow progressors (LTSPs) harbor defective *nef* genes more frequently than rapid progressors infected with subtype B [[17], [18], [19], [20]]. A previous study revealed that Korean Red Ginseng (KRG) ingestion caused a significantly slow decrease in CD4^+^ T cells in patients [21]. Using KRG, some patients can maintain their CD4^+^ T cell counts for more than 20 years without receiving ART. Furthermore, KRG treatment nonspecifically induces gross deletion in *nef* (gΔ*nef*), *gag*, and *pol* in subtype B [20,[22], [23], [24], [25], [26], [27]]. Consequently, it has been reported that the long-term intake of KRG prolongs survival in patients infected with HIV-1 subtype B [28] by decreasing hyper-immune activation [29] and inducing genetic defects [[22], [23], [24], [25], [26], [27]]. However, differing from many reports on subtype B, there are only a few case reports on the gΔ*nef* in patients with non-B infections [30,31].

This study, to our knowledge,is the first report on the proportion of gΔ*nef* in patients with non-B infections and compared the responses with respect to CD4^+^ T cells and the proportion of gΔ*nef* after KRG treatment between patients infected with subtype B and non-B over 10 years. It is likely that an older age and lower amount of KRG in non-B may have played a role in the observed faster annual decrease in CD4^+^ T cells (AD) compared to that found with subtype B infection. However, there was no significant difference in the change in CD4^+^ T cells when the amount of KRG was adjusted. These results of a comprehensive nationwide study suggest the possibility of subtype difference in the genetic stability of HIV-1.

## 2 Materials and methods

### 2.1 Patients

Among our cohort, we selected 77 patients, all of whom were followed for more than 10 years in the absence of ART since diagnosis. Based on phylogenetic analysis of the *nef* gene [32], the non-B subtypes were determined. In this study, the number of patients infected with subtype B and non-B was 46 and 31, respectively (Table 1).

### 2.2 KRG treatment

Among patients with subtype B and non-B infections, 8 and 12 patients were not treated with KRG and 38 and 19 were treated with KRG for a variable period, respectively (Table 2). KRG capsules were manufactured from the roots of 6-year-old, fresh ginseng plants (*Panax ginseng* Meyer) harvested in the Republic of Korea (Korea Ginseng Corporation, Seoul, Korea). One capsule contained 300 mg of powder without any additives. The patients were instructed to take six capsules orally, three times daily for a total daily dose of 5.4 g [[21], [22], [23], [24], [25], [26],^33^]. There was an interruption in KRG ingestion for 4–5 months after the first 6-month pilot study, and there were several other interruptions before July 2000. In addition to KRG, zidovudine was administered to 11 patients in each group (Table 1).

**Table 2:** Comparison of Korean Red Ginseng-treated and control groups among patients infected with human immunodeficiency virus-1 subtype B or non-B subtypes.

Group	Subtype B	Non-B subtypes	P-value
KRG-treated group			
No. of patients	38	19	<0.05
Age at diagnosis (yr)	24 ± 10	31 ± 7	<0.01
Follow-up since diagnosis (months)	174 ± 46	154 ± 40	
Total amount of KRG administered (g)	8236 ± 7276	3929 ± 5849	<0.05
Monthly amount of KRG (g)	47 ± 40	23 ± 25	<0.05
Plasma RNA (copy/mL) at baseline	14,631 ± 19,751	33,073 ± 64,959	
Annual decrease in CD4^+^ T cells (/μL)	26 ± 16∗	40 ± 23	<0.01
**Control group**			
No. of patients	8	12	
Age at diagnosis (yr)	28 ± 5	34 ± 6	<0.05
Follow-up since diagnosis (months)	149 ± 16	146 ± 21	
Plasma RNA (copy/mL) at baseline	7510 ± 5447	18,324 ± 13,236	<0.05
Annual decrease in CD4^+^ T cells (/μL)	41 ± 17∗	52 ± 25	

∗P < 0.05 between 26 ± 16 and 41 ± 17.

KRG, Korean Red Ginseng.

### 2.3 Ethics statement

The institutional review board of the Asan Medical Center approved the protocol of this study (Code 2012–0390; June 4, 2012). All subjects provided their informed consent for inclusion before participating in the study. The study was conducted in accordance with the principles of the Declaration of Helsinki.

### 2.4 Measurement of CD4^+^ T cell count and viral load

CD4^+^ and CD8^+^ T cells were measured using a FACScan flow cytometer (BD, Franklin Lakes, NJ, USA) after staining peripheral blood mononuclear cells with phycoerythrin- and fluorescein isothiocyanate-conjugated antibodies for CD4 and CD8 antigens (Simultest Reagents; BD) [30]. The HIV-1 RNA copy number in the serum was measured using an AMPLICOR 144 HIV-1 monitoring kit (Roche, Basel, Switzerland). The copy numbers were converted into plasma equivalent numbers per mL [34].

### 2.5 RNA preparation, amplification of nef, and subtyping

Total RNA was extracted from 300 μL of serum using a QIAamp UltraSense Viral RNA kit (Qiagen, Hilden, Germany), as described previously [26,32]. The outer primer set (Nef5′5′ and LTR3) and inner primer set (Nef5 and N10) for subtype B and various primer sets for non-B are described in detail in a previous report [32]. The PCR fragments (wild type and short one) were purified from agarose gels with an MG gel extraction SV Kit (MGmed Inc., Seoul, Korea) and sequenced directly using Applied Biosystems 3730XL (Macrogen Inc., Seoul, Korea). PCR contamination was monitored by physical separation for the PCR environment, BLAST searches, and phylogenetic analysis. The subtyping was performed as described in a previous study [32]. Eight out of 46 patients with subtype B infections harbored the western subtype B, whereas the rest harbored the Korean subclade B.

The distribution of non-B (n = 31) was as follows: 13, CRF02_AG; 3, G; 3, A1; 2, A; 2, A2; 2, C; 2, CRF06_cpx; 2 untypable; and 1 each for D, CRF01_AE, and F [32].

### 2.6 Definition of gross deletion in nef

Gross deletion in *nef* was defined as an out-of-frame deletion and a deletion of more than 15 nucleotides [20]. Genes with small in-frame deletions (3–15 bp) that included the last cysteine were considered intact.

### 2.7 Statistical analysis

Data are expressed as the mean ± 2 standard deviations for continuous variables and as counts and percentages for categorical variables. Proportions were compared between phases and groups using the Chi-squared or Fisher's exact tests. Kaplan–Meier survival analyses and Pearson's correlation coefficient were used to explore the difference between subtype B and non-B using MedCalc Statistical Software version 19.2.6 (MedCalc Software Ltd., Ostend, Belgium). Statistical significance was defined as *P* < 0.05.

### 2.8 Sequence data

GenBank accession numbers in this study are as follows: KU588425-857, KY557339-8278, KY683848-995, KX259025-105, MF457421-449, MG461319-39, MG548757-788 and MH396195-369.

## 3 Results

### 3.1 Patient characteristics

The demographic characteristics of the 77 patients infected with HIV-1 subtypes B and non-B are shown in Table 1. The sex ratio was not significantly different between the two groups. The proportions of overseas sailors and age at diagnosis were significantly higher in patients infected with non-B than in those infected with subtype B (*P* < 0.001 for both) (Table 1). The follow-up period since diagnosis was 170 ± 41 and 151 ± 34 months in patients with subtype B and non-B infections, respectively. The number of patients treated with KRG and the amount of KRG administered were significantly higher in subtype B than those in non-B infections (in order, *P* < 0.05 and *P* < 0.01). Consequently, the AD (/μL) was significantly slower in patients with subtype B infections than in those with non-B infections (*P* < 0.001) (Table 1). HIV-1 RNA at baseline was higher in patients with non-B than in those with subtype B infections, despite slightly higher CD4^+^ T cell counts (Table 1). There was no significant correlation between the earliest RNA copy number and AD.

### 3.2 KRG treatment significantly slowed AD in subtype B

**Fig. 1:**
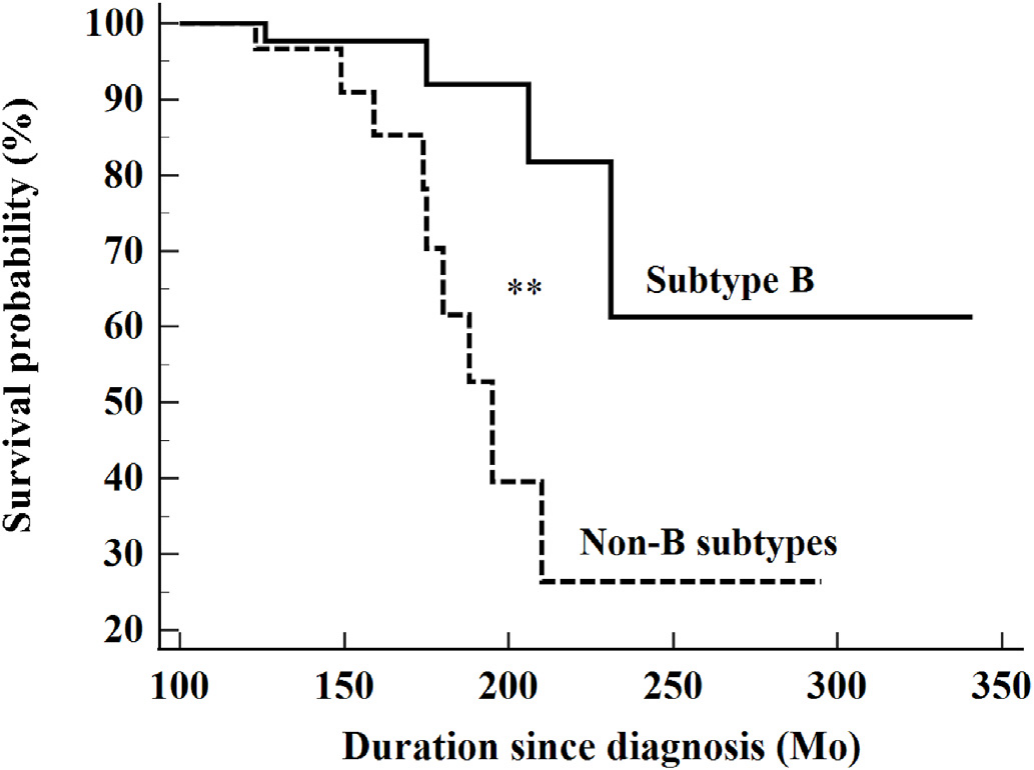
Kaplan–Meier survival analysis showed that the survival probability from diagnosis to death or start of combination antiretroviral therapy was significantly higher in patients with human immunodeficiency virus-1 subtype B (n = 46) than in those with non-B infections (n = 31) (∗∗*P* < 0.01).

In subtype B, the AD was significantly lower in the KRG-treated group than that in the control group (*P* < 0.05), whereas in non-B, there was no such significant difference (Table 2). The number of deaths before ART in the 31 patients with non-B and 46 patients with subtype B infections was 9 (29%) and 4 (8.7%), respectively (*P* < 0.05). Kaplan–Meier survival analysis showed that survival probability was significantly higher in patients with subtype B infections than in those with non-B infections (*P* < 0.01) (Fig. 1). However, there was no significant difference in the AD in the control group, and the difference was less prominent than that in the KRG-treated group (Table 2). This finding suggests that the amount of KRG was significantly related to the difference in the AD.

### 3.3 Correlations between the mKRG, AD, and gΔnef proportion

**Fig. 2:**
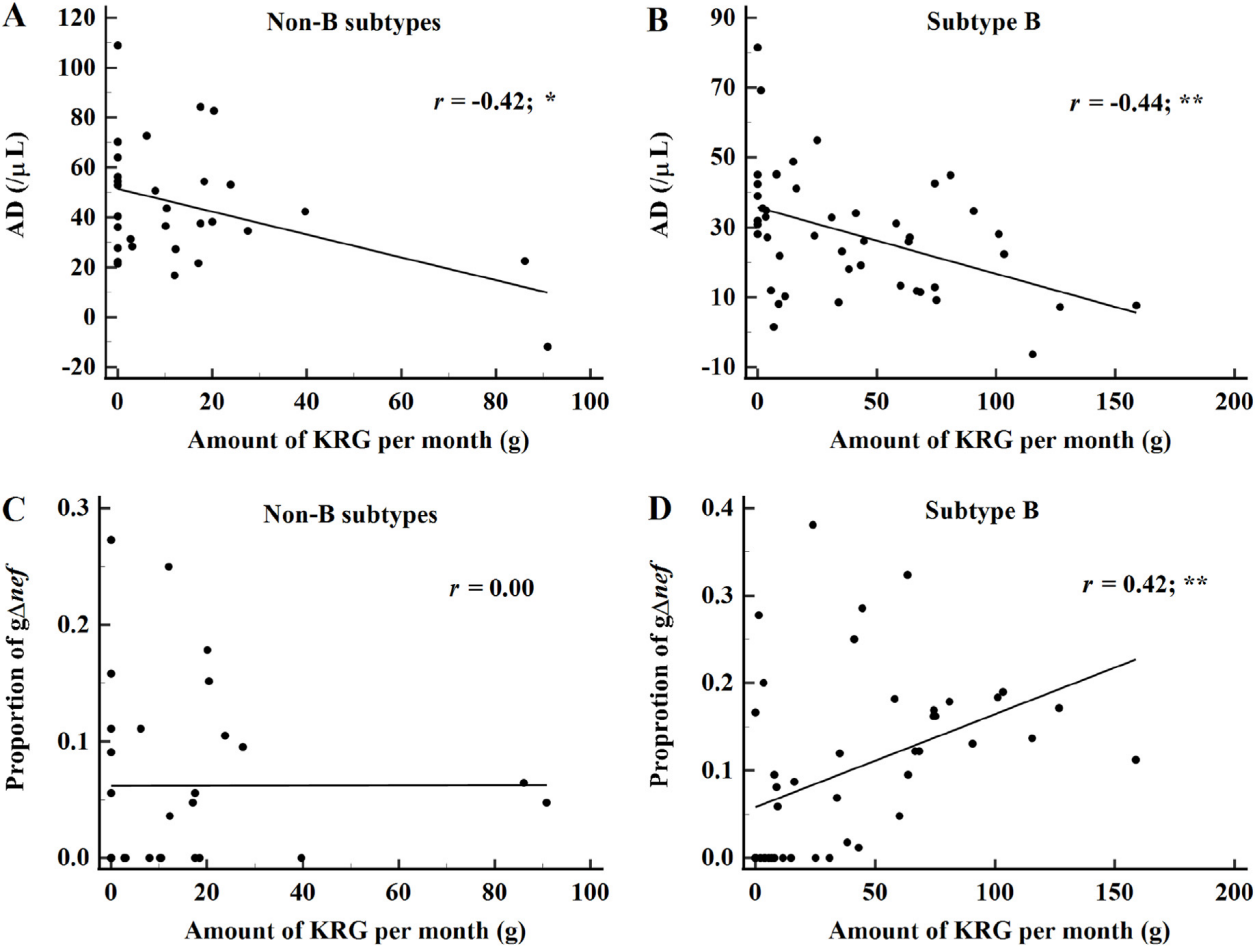
Correlations between the monthly amount of Korean Red Ginseng (mKRG), annual decrease in CD4^+^ T cells (AD), and proportion of gross deletion in the *nef* gene (gΔ*nef*). A significant correlation was observed between mKRG and the AD in both human immunodeficiency virus-1 non-B (A) and subtype B infections (B). A significant correlation between the proportion of gΔ*nef* and mKRG was observed in subtype B, but not in non-B (C). ∗*P* < 0.05 and ∗∗*P* < 0.01.

In both subtypes, there was a significant correlation between mKRG and the AD (*P* < 0.05 for non-B subtypes and *P* < 0.01 for subtype B) (Fig. 2A and B). Interestingly, there was no significant correlation between the mKRG and the proportion of gΔ*nef* in non-B infections (Fig. 2C), whereas there was a significant correlation in subtype B infections (*P* < 0.01) (Fig. 2D).

### 3.4 Comparison of the proportion of gΔnef at 6-month intervals adjusted by the amount of KRG

In subtype B, 38 patients were treated with KRG (Table 1) and the proportion gradually and significantly increased with the increase in KRG treatment from 983 to 1971 g (corresponding to the 7–12 month doses) compared to the baseline (10.3% versus 4.1%; *P* < 0.0001) (Fig. S2). In non-B, 19 patients were treated with KRG (Table 1). Thus, the proportion of gΔ*nef* was significantly higher in subtype B than that in non-B with KRG >1971 g (15.2% versus 6.8% at the amount of KRG >1971 g; *P* < 0.0001) (Fig. S2). Collectively, these results strongly suggest the possibility that non-B may be more biologically stable than subtype B.

### 3.5 Proportion of gΔnef in subtype B was significantly higher than that in non-B

**Fig. 3:**
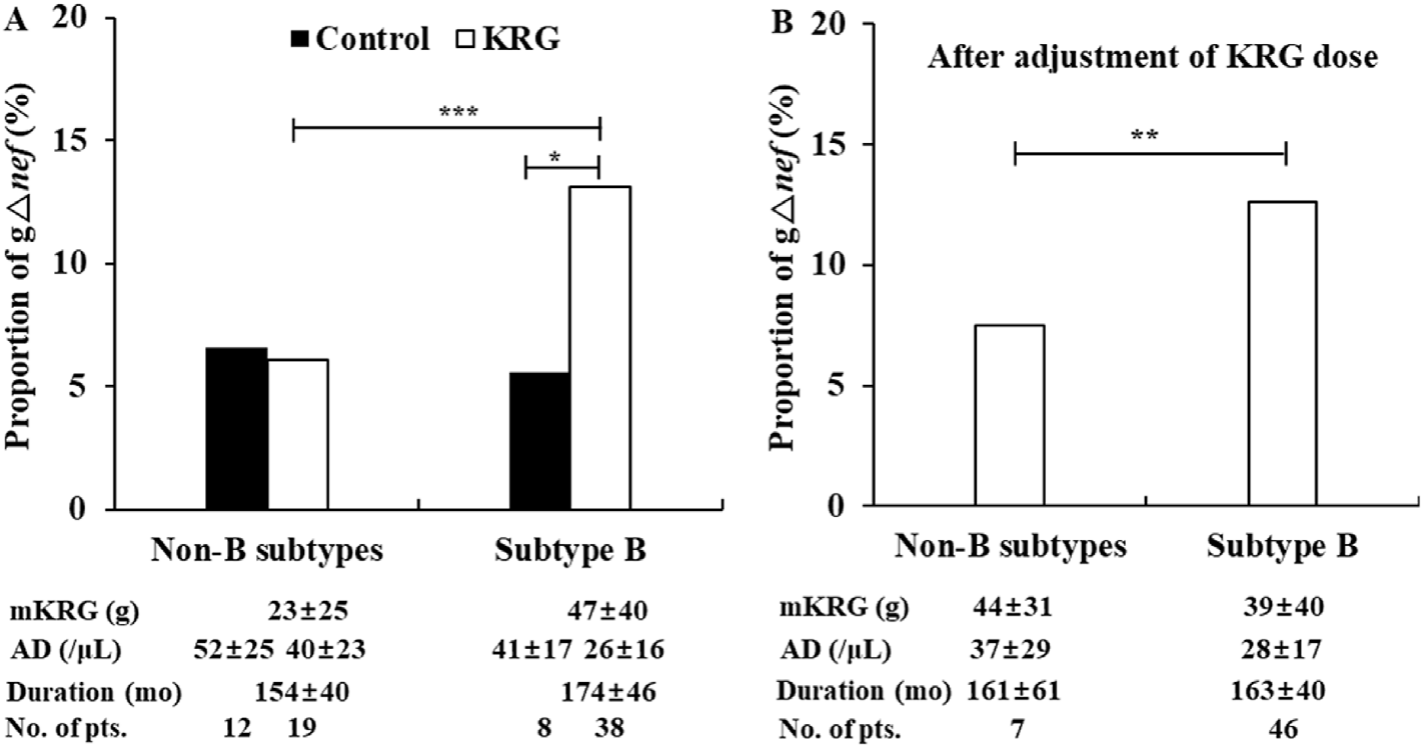
Comparison of the proportion of gross deletion in the *nef* gene (gΔ*nef*) between patients infected with human immunodeficiency virus-1 subtype B and non-B subtypes. (A) In subtype B, the proportion of gΔ*nef* (13.1%) was significantly higher in the Korean Red Ginseng (KRG)-treated group than that in the control group (5.6%). In contrast, no such association was found with non-B infection. (B) Thus, 19 patients with non-B infections were divided into two groups according to the amount of KRG administered per month (mKRG): >20 g (n = 7) and <20 g (n = 12). The mKRG in the former (non-B mKRG >20 g) and in 46 subtype B infected patients was 44 ± 31 g and 39 ± 40 g (Table 1), respectively. After adjustment of the KRG dose, the proportion of gΔ*nef* in the non-B subtypes remained significantly lower (7.5%) than that in the 46 patients infected with subtype B (12.6%) (*P* < 0.01). Duration: from first CD4^+^ T cells to last CD4^+^ T cells before combination antiretroviral therapy; ∗*P* < 0.05, ∗∗*P* < 0.01, and ∗∗∗*P* < 0.0001; AD, annual decrease in CD4^+^ T cells; pts, patients.

We obtained a total of 723 and 1831 *nef* genes in the 31 patients with non-B and 46 patients subtype B infections, respectively, who were followed for >10 years in the absence of ART. In subtype B, the proportion of gΔ*nef* (13.1%; 224/1707) with KRG treatment was significantly higher (overall 2.3-fold) in the KRG group than that in the control group (5.6%; 7/124) (*P* < 0.05) (Fig. 3A), whereas in non-B, the proportion of gΔ*nef* with KRG treatment (6.1%; 36/586) was similar to that in the control group (6.6%; 9/137) (Fig. 3A). Taken together, the proportion of gΔ*nef* with KRG treatment was significantly higher in subtype B than that in non-B (13.1% versus 6.1%) (*P* < 0.0001) (Fig. 3A).

### 3.6 Significantly lower proportion of gΔnef remained in non-B after adjusting the KRG doses

We considered the possibility that the difference in the proportion of gΔ*nef* might be a result of the difference in the amount of KRG (Fig. 3A). Thus, to determine whether the difference in the proportion of gΔ*nef* between non-B and subtype B was associated with the difference in KRG dose, we divided 19 patients with non-B infections into two groups: mKRG >20 g (n = 7; 44 ± 31 g) and ≤20 g (n = 12; 11 ± 6 g). The proportion of gΔ*nef* in the former (mKRG >20 g) was higher (7.5%; 31/413) than that in the latter (2.9%; 5/173) (*P* < 0.05). This finding suggests that the proportion of gΔ*nef* was affected by the KRG dose, even in patients infected with non-B subtypes. The mKRG in the mKRG >20 g group in patients infected with non-B was similar to the 39 ± 40 g mKRG in 46 patients infected with subtype B (Table 1) (Fig. 3B). Despite the similar mKRG in both subtypes, the proportion of gΔ*nef* in non-B (7.5%) was significantly lower than that in subtype B (12.6%) (*P* < 0.01) (Fig. 3B).

The AD was typically higher in non-B (37 ± 29/μL) than that in subtype B (28 ± 17/μL in Table 1) (*P* > 0.05) (Fig. 3B). It was presumed that the significantly lower proportion of gΔ*nef* in non-B compared to that in subtype B might be related to the higher AD in non-B infections*.*

We paid particular attention to three LTSPs with non-B infections and treated with mKRG >20 g. These patients were treated with an mKRG of 72 ± 28 g over 204 ± 78 months (Table 3). Despite a relatively similar AD in patients under the same conditions, the proportion of gΔ*nef* was significantly lower in the three LTSPs (5.1%) than in those with subtype B infections (13.7%) with an mKRG of 68 ± 27 g (*P* < 0.0001) (Table 3).

**Table 3:** Comparison of gross deletion in the *nef* gene in patients intensively treated with KRG.

Group	Subtype B (mKRG >20 g)	3 LTSPs with non-B	P-value
No. of patients (%)	25 (66)	3 (16)	<0.0001
Follow-up since diagnosis (months)	182 ± 50	204 ± 78	
Total amount of KRG administered (g)	11,917 ± 6334	16,272 ± 8936	
Monthly amount of KRG (mKRG)	68 ± 34	72 ± 28	
Annual decrease in CD4^+^ T cells (/μL)	23 ± 14	18 ± 27	
g△*nef* (%)	220/1606 (13.7)	16/312 (5.1)	<0.0001

KRG, Korean Red Ginseng; LTSP, long-term slow progressor.

## 4 Discussion

Our study showed that the proportion of gross deletion in the *nef* gene in patients with subtype B infections was significantly higher than that in patients with non-B subtype infections after KRG treatment. In detail, patients with subtype B infections responded significantly to KRG treatment with respect to AD and the proportion of gΔ*nef.* In contrast, patients with non-B infections responded to KRG treatment with respect to AD, but not gΔ*nef*. In subtype B, the proportion of gΔ*nef* depended on the amount and duration of KRG treatment, whereas in non-B, a similar association to that in subtype B was not observed (Fig. 3). Thus, we believe that this difference might be due to the difference in the amount of KRG provided to the patients harboring the two subtypes (Table 2), although the proportion of overseas sailors and age at diagnosis were significantly higher in the patients infected with non-B than in those infected with subtype B (*P* < 0.001 for both) (Table 1). Even after adjusting for the difference in the KRG dose, the proportion of gΔ*nef* remained significantly lower in non-B than that in subtype B (Fig. 3B) (*P* < 0.01). Our data suggest that this difference in the proportion of gΔ*nef* is related to the difference in AD between the two subtypes; furthermore, the genetic defect in *nef* appears to be one of several factors supporting long term non-progressors infected with subtype B.

The difference in the proportion of gΔ*nef* between the two subtypes can be explained by the following factors: first, there was a low viral load of <100 copies/mL for 8–10 years in patients 88–17 and 90–14 and approximately 1000 copies/mL for 10 years in patient 93–01 (Fig. S1). As the proportion of gΔ*nef* is typically less than 10% of the viral populations of the wild type, it was more difficult to detect a minor portion of viruses by PCR in these three patients with a low viral concentration. This phenomenon was also observed during suppressive ART compared to that obtained before ART (20.6% versus 3.2%; 15.6% versus 2.9%) [24,26]. In addition, the number of *nef* from these three patients (n = 312) comprised 53% of the number of non-B *nef*. Moreover, we found the same phenomenon in *pol*; the proportion of genetic defects was also significantly higher in subtype B (11.9%) [27] than that in the same three patients with non-B (1.9%) (*P* < 0.01), with a significant decrease in the proportion of gΔ*pol* during ART (11.9% versus 4.1%) [27]. Second, there were differences in the passage number over time between the two subtypes in the Korean population. In detail, the transmission of subtype B occurred actively, particularly in men who have sex with men among Koreans, whereas the transmission of non-B occurred in spouses via heterosexual contact [32]. Thus, this subtype is relatively less adapted to Koreans, as shown in a previous study [35]. Third, regarding multiple passages over time, the replicative fitness of HIV-1 may have decreased since the start of the pandemic [36]. This attenuation in the Korean subclade of HIV-1 subtype B might be a consequence of serial bottlenecks during transmission and the increase in sequence length over time [37], resulting in the adaptation of HIV-1 to the human host [13].

Many reports have suggested that long term non-progressors harbor gΔ*nef* more frequently than progressors [[17], [18], [19]]. The frequency of gΔ*nef* is very rare even in long term non-progressors infected with subtype B [19]. There are only a few case reports on gΔ*nef* in patients with non-B infections [30,31]. In addition, except for a patent on a method of deleting *nef* in HIV-1 using red ginseng (No. 20072033123, 2009, Australia) [20], there are no similar reports on any therapeutic agent, including medicinal food, that deletes or attenuates microorganism or virus similar to gross deletions in HIV-1 such as gΔ*nef* [26].

Regarding the mechanism underlying the occurrence of gΔ*nef*, we propose two potential pathways. First, g△*nef* might result indirectly from “immunological pressure” such as anti-inflammatory action, immune modulation toward Th1 cytokines, and potentiation of cytotoxic T lymphocyte activity by viral suppression [26]. Second, due to this immunological pressure on proviral DNA within the host chromosome, many cellular factors could be involved in provirus latency [26]. It is well known that chromatin remodeling enzymes such as histone deacetylases (HDACs) recruited to the HIV promoter play an important role in HIV latency. HDAC inhibitors might lead to the activation of HIV in latently infected cells and result in the fragmentation of proviral DNA. Recently, ginsenosides Rg3, Rh2, and compound K have been established as HDAC 3 inhibitors [[38], [39], [40]]. In addition, approximately 200 substances, such as ginsenosides, polysaccharides, polyacetylenes, peptides, trace elements, and amino acids have been isolated from ginseng. Therefore, it might be difficult to elucidate the exact mechanism, although a previous study found that ribonuclease extracted from *P. ginseng* displays an inhibitory activity against HIV-1 reverse transcriptase [41].

The current study had several limitations. First, there was a significantly higher proportion of overseas sailors and patients of older age in the non-B group compared to that in the subtype B group. Second, patients with non-B infections were administered a lower amount of KRG. Third, the mode of transmission was different between the two subtypes. Fourth, the viral load at baseline was higher in patients with non-B infections than in those with subtype B infections.

The findings of this study help recognize the potential difference in genetic stability between subtype B and non-B under KRG treatment for an extended period. Further study is needed to clarify how much the low proportion of gΔ*nef* in non-B is associated with the AD in patients infected with the rapidly progressing subtype D.

## Declaration of competing interest

The authors declare no conflicts of interest.
