# Supplementary figures and images for: Impact of HIV-1 subtypes on gross deletion in the nef gene after Korean Red Ginseng treatment
Source: J Ginseng Res. 2022 Feb 26;46(6):731–7. doi: 10.1016/j.jgr.2022.02.005 (PMC9597433; doi:10.1016/j.jgr.2022.02.005)

| **CD4+ T cell (/uL) (●––––●)** | 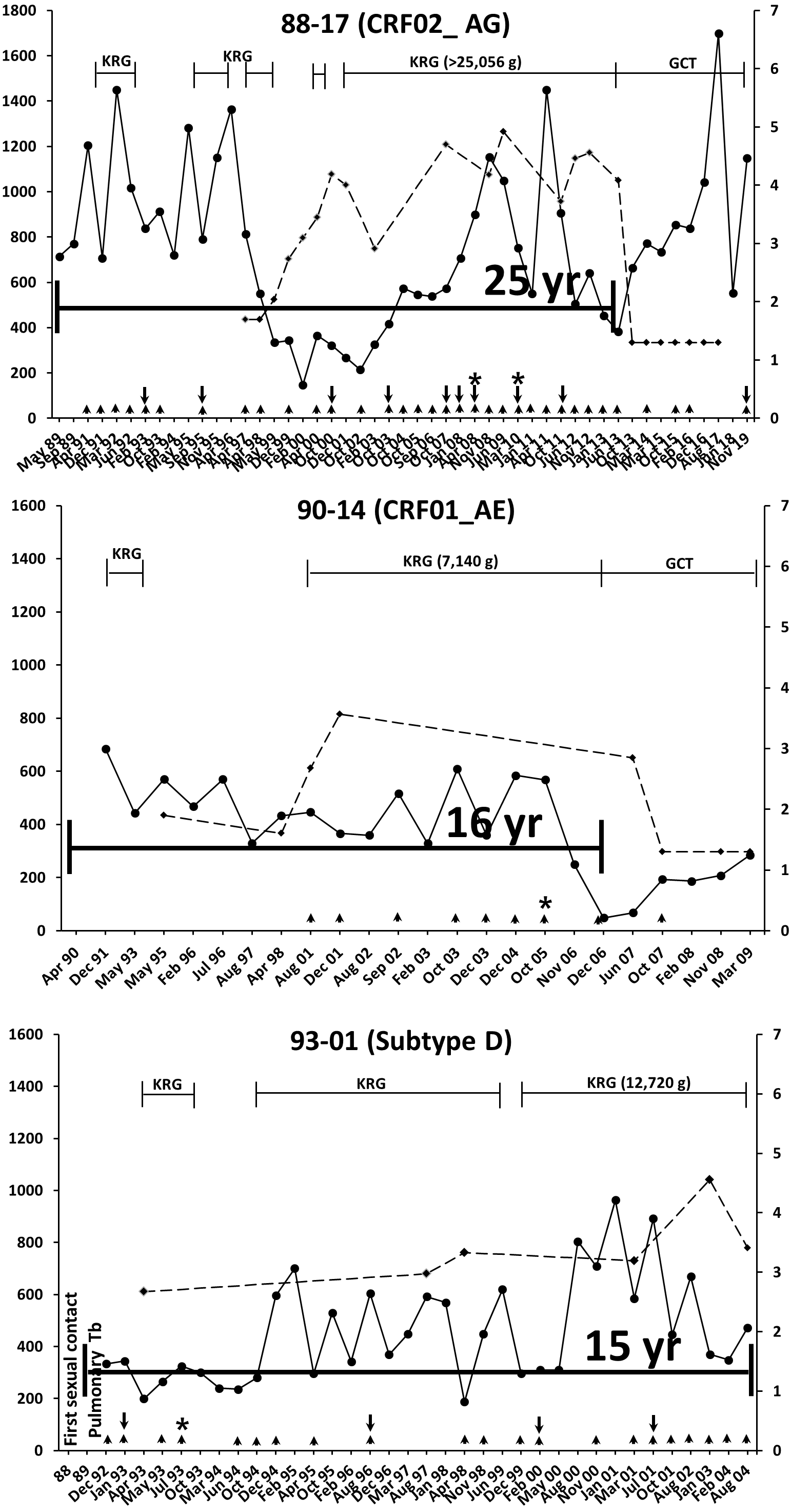 | **RNA copy (log/mL) (◆－－－◆)** |
| --- | --- | --- |
|  | **Sampling date** |  |

Fig. S1.

Supplement: Multimedia component 1 [file mmc1.docx]

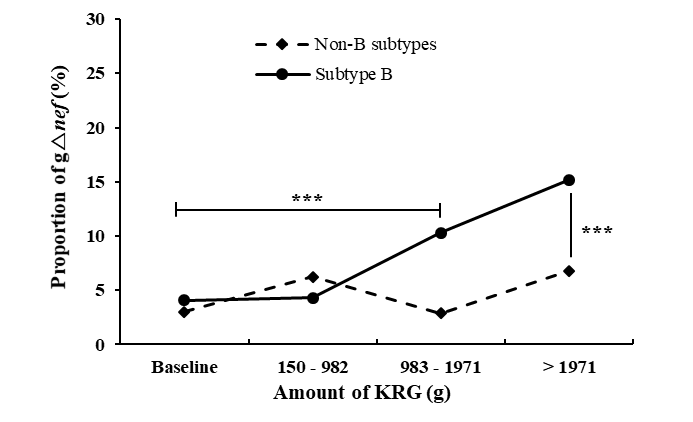


**Fig. S2**

Supplement: Multimedia component 2 [file mmc2.docx]
